# The Role of Genetic Variants of Stromal Cell-Derived Factor 1 in Pediatric HIV-1 Infection and Disease Progression

**DOI:** 10.1371/journal.pone.0044460

**Published:** 2012-09-04

**Authors:** Ketty Gianesin, Riccardo Freguja, Francesco Carmona, Marisa Zanchetta, Paola Del Bianco, Sandro Malacrida, Marco Montagna, Osvalda Rampon, Carlo Giaquinto, Anita De Rossi

**Affiliations:** 1 Section of Oncology and Immunology, AIDS Reference Center, Department of Surgery, Oncology and Gastroenterology, University of Padova, Padova, Italy; 2 Istituto Oncologico Veneto – IRCCS, Padova, Italy; 3 Department of Neurosciences, University of Padova, Padova, Italy; 4 Department of Pediatrics, University of Padova, Padova, Italy; University of Texas Health Science Center/South Texas Veterans Health Care System, United States of America

## Abstract

Stromal cell-Derived Factor 1 (SDF1) is the natural ligand of CXCR4, the coreceptor of HIV-1 X4 viruses. This study investigated the role of the single nucleotide polymorphism (SNP) rs1801157 (NM_000609.5:c.*519G>A) of the *SDF1* gene in the natural history of mother-to-child transmission of HIV-1 and disease progression of HIV-1-infected children. The study was conducted in 428 children born to HIV-1-seropositive mothers, who had not undergone antiretroviral therapy (ART) during pregnancy, and in 120 HIV-1-infected children for whom the end-point was the onset of AIDS or the initiation of ART; 16 children developed early AIDS (<24 months of life), 13 from 24 to 84 months of age, and 14 had late AIDS (>84 months). The rs1801157 SNP was not associated with risk of perinatal infection in any genetic models tested. By contrast, this SNP influenced disease progression in a time-dependent manner. rs1801157 GA heterozygous children had a higher risk of late AIDS (HR = 6.3, 95%CI 1.9–20.7, p = 0.002) than children with the rs1801157 GG genotype. Children were studied for viral coreceptor usage at birth, after 84 months of age and/or at AIDS onset. While R5 viruses using CCR5 coreceptor were predominant at birth (94%) and at early AIDS (85%), viruses using CXCR4 coreceptor emerged during the course of infection and were detected in 49% of children older than 84 months and in 62% of late AIDS. The rs1801157 SNP did not influence the emergence of R5X4 viruses, but children with the rs1801157 GA genotype and R5X4 viruses were at significantly higher risk of late AIDS than children with rs1801157 GG genotype (OR = 8.0, 95% CI 1.2–52.2, p = 0.029). Our results indicate that the rs1801157 SNP does not influence perinatal infection, but impacts disease progression. This effect is time-dependent and linked to the coreceptor-usage of viral variants that undergo evolution during the course of HIV-1 infection.

## Introduction

The interaction of viral and host factors is believed to determine not only the risk for initial Human Immunodeficiency Virus type-1 (HIV-1) acquisition, but also the course of the infection. Increasing data support host genetic factors as important determinants of HIV-1 infection and pathogenesis.

The chemokine receptors CXCR4 and CCR5 have been identified as the major coreceptors for HIV-1 entry into target cells [1]–[4]. Mother-to-child transmission (MTCT) is the main source of pediatric HIV-1 infection. Viruses using CCR5 (R5 viruses) are the predominant viral variants transmitted from mother to child and detected in the newborn [5], [6]. Evolution of HIV-1 coreceptor usage has been demonstrated during disease progression in adults as well as in children [7], [8]. The evolution usually involved change from CCR5 usage to CXCR4 usage alone (X4 viruses) or in combination with CCR5 (R5X4 viruses). The switch from R5 phenotype to X4 or R5X4 phenotype, which occurred in approximately 50% of infected individuals [8], [9], was associated with accelerated CD4 T cell decline and progression to AIDS [10], [11].

Genetic polymorphisms of chemokine receptors and related ligands have been demonstrated to influence both HIV-1 transmission and disease progression [12]. The deletion of 32 bp (Δ32) in the *CCR5* gene impairs the expression of coreceptor on the cell membrane and Δ32 homozygosity confers resistance to infection by R5 viruses [13]. Heterozygotes express low levels of CCR5 receptors, slowing HIV-1 replication, spread and pathogenesis [14], [15]. Furthermore, nucleotide variants in the *CCR5* promoter impact disease outcome; homozygosity for P1 haplotype is associated with rapid progression to AIDS in adults as well as in children [16], [17].

The chemokine Stromal cell-Derived Factor 1 (SDF1) is the unique natural ligand of the CXCR4 receptor, and it may interfere with HIV-1 infection [18], [19]. SDF1 exhibits genetic polymorphisms, including the single nucleotide polymorphism (SNP) rs1801157 (NM_000609.5:c.*519G>A, also known as SDF1 3′G>A). This SNP is located in the 3′ untranslated region of the gene that may serve as a target for cis-acting factors, thus influencing the chemokine’s expression [20], [21]. This SNP may thereby influence mRNA stability; *SDF1* mRNA containing the 3′A variant has a two-fold longer half-life compared to the 3′G variant [22]. Studies in adults have suggested that rs1801157 AA homozygosity was associated with increased plasma levels of SDF1 [23] and delayed progression to AIDS [24], [25]. However, these findings were not confirmed by others [26], [27]. Moreover, several studies suggested an association between the rs1801157 AA genotype and accelerated disease progression [28]–[30].

With regard to pediatric HIV-1 infection, there are very few data. One study in African population indicated that the mother’s, but not the child’s, rs1801157 GA genotype increases the risk of perinatal transmission of HIV-1, particularly during breastfeeding [31], but this association was not confirmed in breastfeeding mothers receiving zidovudine [32]. Few studies suggested that the rs1801157 AA genotype was associated with accelerated disease progression, but this variant occurred in a very small fraction of subjects [33], [34]. Moreover, the protective effect of *CCR5*Δ32 heterozygosity appeared to be abrogated by the rs1801157 A allele [35]. None of these studies addressed the role of the rs1801157 genotype in relation to viral coreceptor usage at the onset of AIDS.

The aim of this study was to evaluate the role of *SDF1* rs1801157 polymorphism in the natural history of perinatal infection and disease progression of HIV-1-infected children, in relation to the coreceptor usage of the infecting viruses and time of AIDS onset.

## Methods

### Patients

The study population for the assessment of rs1801157 SNP in MTCT included 428 children (148 HIV-1-infected and 280 HIV-1-uninfected children), born to HIV-1-seropositive mothers between 1984 and 2002, whose virological analyses for diagnosis of HIV-1 infection were conducted at the AIDS Reference Center of Padova University. Inclusion criteria were the known HIV-1-seropositive status of the mother at delivery, and the absence of antiretroviral prophylaxis during pregnancy and/or at delivery. No child was breastfed. Diagnosis of HIV-1 infection was performed by virus isolation and polymerase chain reaction (PCR) [36], [37]. The study population for the assessment of the rs1801157 SNP in disease progression involved 120 HIV-1-infected children followed from birth at the Pediatric Department of Padova University. Clinical and immunologic staging of the HIV-1-infected children was defined according to the classification system of the Centers for Disease Control and Prevention (CDC) [38].

The end-point of the study was the onset of severe clinical manifestations (stage C, CDC), or the initiation of antiretroviral therapy (ART). The median follow-up was 88 (interquartile range [IQR] 36-135) months. According to the natural history of pediatric HIV-1 infection [39]–[41], children were classified as early progressors if they developed severe symptoms of disease within two years, and late progressors if they developed severe symptoms of disease after seven years of age. The study was performed in accordance with the Helsinki Declaration and was approved by the Ethics Committee of the Istituto Oncologico Veneto, Prot. 2131, 2012/04 and Azienda Ospedaliera Padova, 30/01/2012. An informed verbal consent was obtained from the parents or the guardians of the children by a pediatrician and recorded on the clinical notes. According to the national law (General Authorization for the genetic data - Garante della Privacy 24 June 2011,G.U. no.159, 2011) the written consent was not required for this study. The Ethics Committees verified the requirements and approved this consent procedure.

### SDF1 Genotyping

Genomic DNA was extracted from peripheral blood mononuclear cells (PBMC) with the QIAmp DNA Blood mini kit (Qiagen, Hilden, Germany), according to the manufacturer’s instructions. Polymorphic sites in genomic DNA were analyzed by the TaqMan allelic discrimination assay. Primers for genotyping the rs1801157 SNP were F5′-CAAGCCTAGTGAAGGCTTCTCTC-3′ and R5′-TCAGGGTAGCCCTGCTGC-3′. The probes were as follows (allele-specific nucleotides are underlined): 5′-FAM-TGGGAGCCGGGTCTGCCTCT-BHQ1-3′ and 5′-HEX-ACATGGAGCCAGGTCTGCCTCTT-BHQ1–3′. PCR was performed in a thermal cycler (LightCycler 480, Roche Diagnostic, Mannheim, Germany) in a reaction volume of 20 µl containing 600 nM of each primer, 300 nM of probe for SNP G, 100 nM of probe for SNP A, 10 µl of 2x LightCycler 480 Probe Master (Roche Diagnostic) and 2 ng of sample DNA. The cycling conditions were 95°C for 10 min, followed by 45 cycles, each of 95°C for 10 sec, 62°C for 1 min and 72°C for 1 sec. Allele discrimination and genotype determination were based on the endpoint fluorescence measured by the LightCycler 480 detection system. Three positive controls, one for each of the possible genotypes, were included. Accuracy of genotyping was confirmed by direct sequencing of randomly selected samples, as previously described [42] using the same primers used in TaqMan assay.

### CCR5 Genotyping

The P1/P1 haplotype on the promoter of the *CCR5* gene was determined by heteroduplex analysis, as described previously [17].

### Viral Phenotype Analysis

Primary isolates were obtained by culturing PBMC from each subject with phytohemagglutinin (PHA)-stimulated PBMC from healthy donors, exactly as previously described [5]. Primary isolates were available from 53 children at birth, from 43 of 49 children older than 84 months of age, from 13 of 15 early AIDS and 13 of 14 late AIDS cases. Coreceptor usage of primary isolates was determined by viral infection in U87.CD4 cells that stably expressed CCR5 or CXCR4 coreceptors, as previously described [5]. On days 5 and 8, supernatants were collected and tested for p24 antigen using a commercially available assay (Vironostika® HIV Ag/Ab, Biomérieux SA, France). The laboratory strains HIV_BAL_ (R5-tropic) and HIV_IIIB_ (X4-tropic) were used as controls in all *in vitro* experiments.

### V3 Genotyping

HIV-1 RNA was extracted from frozen plasma samples with the QIAmp RNA Viral Mini Kits (Qiagen), according to the manufacturer’s instructions, and reverse transcribed by SuperScript III RT (Invitrogen, Milan, Italy) and random primers (Invitrogen). The cDNA obtained was used for nested PCR amplifications to sequence the *V3* region. The *V3* loop was amplified with the outer primer pair V3A (5′-TACAATGTACACATGGAATT-3′) and V3D (5′-ATTACAGTAGAAAAATTCCCC-3′) and the inner primers V3B (5′-TGGCAGTCTAGCAGAAGAAG-3′) and V3C (5′-CTGGGTCCCCTCCTGAGG-3′). The first-round PCRs were performed with 5 ul of cDNA, in a 50 ul reaction mixture containing 5 ul of 10X PCR Buffer II (Applied Biosystems, Foster City, CA, USA), 700 nM of each of the outer primers, 200 nM of deoxyribonucleotide triphosphates, 1.6 mM of MgCl_2_, and 1.5 U of AmpliTaq DNA polymerase (Applied Biosystems). Forty cycles were carried out in a thermal cycler (2720 thermal cycler, Applied Biosystem), each consisting of 30 sec at 94°C, 30 sec at 54°C, and 1 min at 72°C, followed by one cycle at 72°C for 7 min. Five microliters of the first-round PCR were amplified in a nested PCR with 100 uM of the inner primers; 40 cycles were run, each consisting of 30 sec at 94°C, 30 sec at 59°C, and 1 min at 72°C, followed by a final extension at 72°C for 7 min. All negative controls from the first round of amplification were included in the second amplification step. The amplified products were separated on a 2.5% agarose gel. Direct sequencing was performed as described previously [42], using the V3B and V3C primers. The sequence alignments were subsequently inspected manually. HIV-1 tropism was predicted from the *V3* genotype using position-specific scoring matrices PSSM_X4/R5_ [43] (http://fortinbras.us/cgi-bin/fssm/fssm.pl). Standard sequencing with PSSM values below the predetermined cutoff of -6.96 were called R5, whereas those with scores greater than or equal to -6.96 were defined as R5X4 [43]. Where sequence ambiguity to the presence of nucleotide mixtures occurred, the permutation with the highest PSSM score was used to assign the score for a given replicate, to increase the sensitivity for detection of X4 variants [44]. For each HIV-1 RNA sample, the *V3* sequence with PSSM results were compared with the results of the viral phenotype assay. For discordant results, the test with the broadest prediction was considered.

### Statistical Analyses

Genotype and allele frequencies, and Hardy-Weinberg equilibrium tests of the rs1801157 SNP were performed separately among cases and controls using the SNPStats program [45]. Estimation of the power of the study to detect association between SNP and HIV-1 infection was evaluated using the genetic power calculator available at http://pngu.mgh.harvard.edu/~purcell/gpc/for minimum detectable risk calculations using SNP effects under different study constrains. The minimum detectable effects with odds ratio (OR) was calculated at various allele frequencies of the SNP allele in a codominant model, based on an alpha of 0.05 and a prevalence 1/1.000 in the general population. With this sample size, we had 80% power to detect an OR of 4.3 if the risk allele frequency was 35%. Unconditional logistic regression analysis was performed with SNPStats to investigate the association between the SNP genotype and infection status. Because the mode of inheritance was unknown, to increase the power of the study four genetic models (codominant, dominant, recessive and overdominant) were considered and OR and 95% confidence intervals (CI) were calculated.

The probability of acquiring disease was calculated with the Kaplan-Meier method and the log-rank test was used to test for differences between genotype categories. Hazard ratios (HR) and their 95% CI based on the Cox proportional hazard model were estimated to test the association between genotypes, and risk of stage C. The assumption of proportional risk was evaluated using the Schoenfeld residuals test, which was significant for the rs1801157 (p = 0.03). To take into consideration this time-dependent effect, we added to the basic Cox proportional hazard model an interaction of the rs1801157 with a heaviside step function to get constant hazard ratios for different time intervals [46]. The function of risk was the following:

h(t, SDF1) = h_0_(t) exp(β_1_×SDF1+ γ_1_×SDF1×g(t)), where g(t) = 0 if t≤t_0_ and g(t) = 1 if t >t_0,_ from which we obtain two different HR for the variable rs1801157: HR(SDF1) = exp(β_1_) if t≤t_0,_ and HR(SDF1) = exp(β_1_+ γ_1_) if t>t_0._ We chose visually the change point, t_0,_ as the integer year value were the two survivor curves appeared to diverge. OR and 95% CI were calculated to estimate association between the rs1801157 genotype and viral coreceptor usage. All tests were two-sided, and a p-value less than 0.05 was considered statistically significant. Statistical analyses were performed using Epi Info™ 3.5 and the SAS version 9.1.3 (SAS Institute, Cary, NC).

## Results

### Relationship between the rs1801157 SNP and Risk of Pediatric HIV-1 Infection

To analyze the impact of the rs1801157 SNP on HIV-1 perinatal infection, 148 HIV-1-infected and 280 exposed HIV-1-uninfected children were analyzed according to different genetic models. Genotype and allele frequencies for the rs1801157 were in Hardy-Weinberg equilibrium in both groups and were in agreement with frequencies reported in the NCBI database on the white population (data not shown). The rs1801157 AA genotype was very rare in both HIV-1-infected [3 (2.0%) of 148 children] and HIV-1-uninfected children [9(3.2%) of 280 children]. Forty-four (29.7%) of 148 HIV-1-infected and 97(34.6%) of 280 HIV-1-uninfected children were heterozygous for the rs1801157 A allele. The SNP showed no significant association with the risk of HIV-1 infection in any genetic model considered (Table 1). Values of maternal viral load at delivery were available in a subgroup of 139 mothers (median log_10_ 3.74, range log_10_ 1.60-6.32, HIV-1 RNA copies/ml of plasma). After adjustment for maternal viral load, the rs1801157 SNP was found to not be significantly associated with the risk of HIV-1 infection.

**Table 1:** Frequencies of the rs1801157 genotype and risk of pediatric HIV-1 infection.

Genetic model	rs1801157genotype	Uninfectedchildren, N. (%)	Infectedchildren, N. (%)	OR(95% CI)	p-value
Codominant	GGGAAA	174 (62.1%)97 (34.6%)9 (3.2%)	101 (68.2%)44 (29.7%)3 (2.0%)	1.000.78 (0.51–1.20)0.57 (0.15–2.17)	0.410
Dominant	GGGA + AA	174 (62.1%)106 (37.9%)	101 (68.2%)47 (31.7%)	1.000.76 (0.50–1.16)	0.210
Recessive	GG + GAAA	271 (96.8%)9 (3.2%)	145 (98.0%)3 (2.0%)	1.000.62 (0.17–2.34)	0.470
Overdominant	GG + AAGA	183 (65.4%)97 (34.6%)	104 (70.3%)44 (29.7%)	1.000.80 (0.52–1.23)	0.300

### Relationship between the rs1801157 SNP and Disease Progression

**Figure 1:**
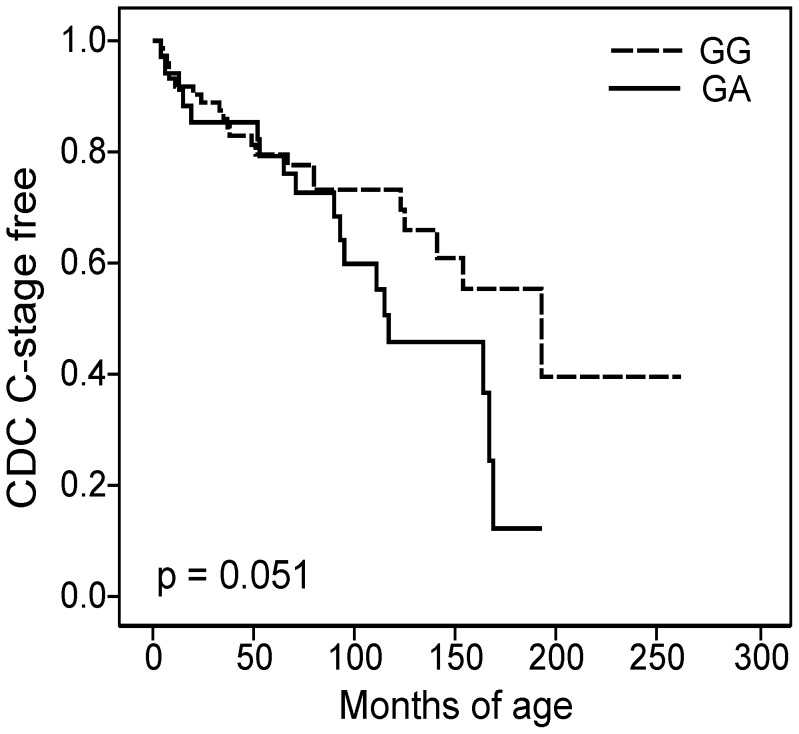
Kaplan-Meier curves showing the relationship between rs1801157 genotype and the onset of stage C CDC. The dashed line represents children with the GG genotype; the black line represents children with the GA genotype.

The relationship between the rs1801157 SNP and disease progression was studied in 120 HIV-1-infected children followed from birth. The end-point of the study was the onset of severe clinical manifestations (stage C, CDC) or the initiation of ART. Nine children were excluded from the study because of initiation of early ART within three months of age. Of the 111 children, seventy-seven children (69.4%) had the rs1801157 GG genotype and among them 24 (31.2%) developed AIDS. Thirty-two children (28.8%) had the rs1801157 GA genotype and 18 (56.3%) progressed to AIDS. Overall, the rs1801157 GA genotype tended to be associated with a worse prognosis and a rapid disease progression, with both Kaplan-Meier (p = 0.051) (Figure 1) and Cox analyses (HR = 1.83, 95% CI 0.99-3.41; p = 0.055) (Table 2). Children with the rs1801157 GA genotype had a median time to stage C of 114 (92;-) months *versus* children with the rs1801157 GG genotype who had a median time to stage C of 199 (140;-) months. Only 2 children had the rs1801157 AA genotype; one developed AIDS at 5 months and the other one entered ART at 135 months. These cases were excluded from the analysis, because there was not sufficient statistical power to evaluate the effect of rs1801157 AA homozygosity.

**Table 2:** rs1801157 genotypes and risk of disease progression according to different time period.

Time periodage in months	rs1801157genotype	AIDS/N.	HR(95% CI)	p-value
**Overall**	GGGA	24/7718/32	1.001.83 (0.99–3.41)	0.055
**0-24 months**	GGGA	10/775/32	1.001.18 (0.40–3.44)	0.768
**25–84months**	GGGA	9/634/27	1.000.89 (0.27–2.87)	0.837
**>84 months**	GGGA	5/319/18	1.006.32 (1.93–20.72)	0.002
**<84 months**	GGGA	19/779/32	1.001.03 (0.47–2.28)	0.944
**>84 months**	GGGA	5/319/18	1.006.32 (1.93–20.72)	0.002

Interestingly, the effect of the rs1801157 SNP seemed to be time-dependent, with survival curves beginning to diverge at approximately 90 months of age, suggesting a change in the proportionality of risk (Figure 1). To better investigate this aspect, we divided the time periods into distinct intervals and fitted the proportional hazard models in each interval [46]. To determine the boundaries of the time intervals, we selected periods according to the natural history of pediatric HIV-1 infection [39]–[41] and the divergence of survival curves. Among children with the GG or GA genotype, 15 developed AIDS within the first 24 months of life (early AIDS), 13 developed AIDS between 24 and 84 months of age, and 14 developed AIDS after 84 months of age (late AIDS). Cox analyses revealed that the influence of the rs1801157 SNP was not relevant before 84 months, while it became strongly significant after 84 months of age: the rs1801157 GA genotype was associated with a higher risk of late AIDS than the rs1801157 GG genotype (HR = 6.32, 95% CI 1.93–20.72, p = 0.002) (Table 2). The same evidence emerged when analysis was performed considering two time intervals, before and after 84 months of age (Table 2).

Five infants in our cohort were Δ32 heterozygous in the *CCR5* gene. After adjustment for *CCR5*Δ32, the rs1801157 GA genotype did not influence the onset of AIDS before 84 months (HR = 0.74, 95% CI 0.30–1.30, p = 0.506), but remained significantly associated with a higher risk of late AIDS onset (HR = 15.79, 95% CI 2.56–97.15, p = 0.003).

### Analysis of Viral Coreceptor Usage

**Figure 2:**
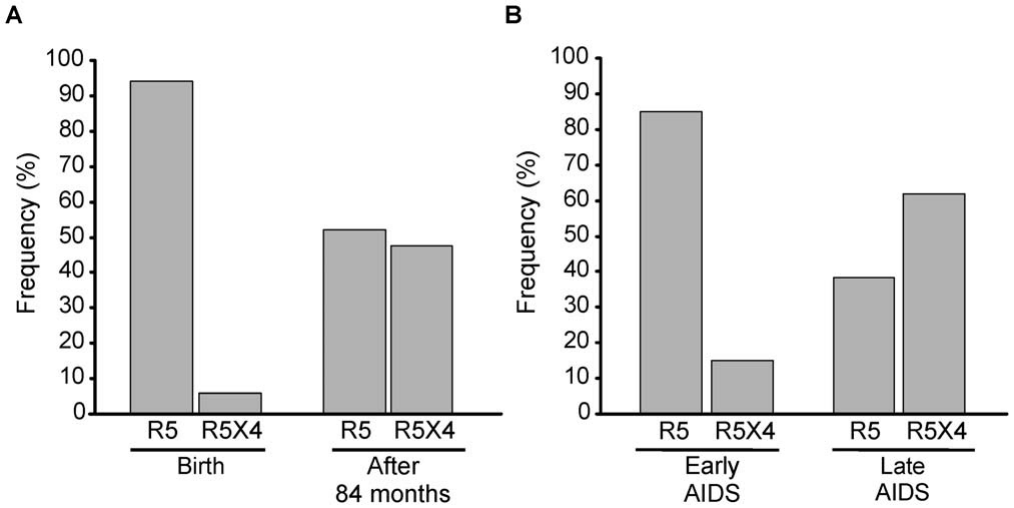
Viral coreceptor usage distribution in HIV-1-infected children. R5 and R5X4 viral strains in (A) HIV-1-infected children before and after 84 months of age; (B) at the onset of early AIDS (before 24 months of age) and late AIDS (after 84 months of age).

Considering the different impact of the rs1801157 SNP in relation to the time of infection and taking into account that coreceptor usage of infecting viruses may evolve during the course of infection, we analyzed the viral coreceptor usage in all children for whom primary isolates were obtained at birth, after 84 months of age and/or at AIDS onset. At birth, primary isolates were available from 53 children, 34 of 77 (44.2%) with the rs1801157 GG genotype and 19 of 32 (59.4%) with the rs1801157 GA genotype; 50 of 53 (94%) primary isolates had an R5 phenotype (Figure 2A) and R5 viral strains were also predominant in early AIDS (11 of 13 analyzed cases; 85%) (Figure 2B). Viruses using the CXCR4 coreceptor emerged during the course of infection; and were detectable in 21 out of 43 (49%) children analyzed after 84 months of age (Figure 2A) and in the majority of late AIDS (8 of 13 analyzed cases; 62%) (Figure 2B). Overall, R5X4 viruses tended to be associated with an increased risk of AIDS (HR = 2.00, 95% CI 0.90–4.40; p = 0.088), but early AIDS was significantly more associated with R5 viruses than late AIDS (Fisher test p = 0.041).

The rs1801157 SNP was not associated with emergence of CXCR4-usage viral strain; indeed, after 84 months of age, R5X4 viruses were found in 56% (9 of 16) and in 44% (12 of 27) of children with the rs1801157 GA and GG genotype, respectively (Fisher test p = 0.537).

### Association between the rs1801157 Genotype, *CCR5* Haplotype, Viral Coreceptor Usage and Disease Progression

Association analysis confirmed that the rs1801157 SNP was not related to early AIDS (p = 0.716), but strongly associated with late AIDS; children with rs1801157 GA genotype had a higher risk of late AIDS (OR = 5.20, 95% CI 1.38–19.67, p = 0.015) than children with the rs1801157 GG genotype (Table 3). This finding suggested an interaction between the rs1801157 SNP and viral coreceptor usage. This was supported by analysis of the *CCR5* promoter haplotype. In agreement with previous data [17], children carrying the P1/P1 haplotype showed a higher risk of developing early AIDS than children carrying other *CCR5* haplotypes (P1/P4, P2/P2, P2/P4, P4/P4). As opposed to that observed with the *SDF1* SNP, the effect of the P1/P1 *CCR5* haplotype was strongest in early AIDS (OR = 8.48, 95% CI 2.30–31.29; p = 0.001) when R5 viruses prevailed and lost after 84 months of age when R5X4 viral variants emerged in half of the studied children (Table 3).

**Table 3:** Association between the rs1801157 genotype, *CCR5* haplotype and early and late AIDS.

	Genotype	OR (95% CI)	p-value
**Early AIDS**			
SDF1rs1801157CCR5Haplotype	GGGAP1/P1others*	1.001.24 (0.39–3.97)8.48 (2.30–31.29)1.00	0.7160.001
**Late AIDS**			
SDF1rs1801157CCR5Haplotype	GGGAP1/P1others*	1.005.20 (1.38–19.67)0.78 (0.07–8.52)1.00	0.0150.837

* Others: CCR5 haplotypes P1/P4, P2/P2, P2/P4, P4/P4.

Analysis of the association between the rs1801157 genotype, viral coreceptor usage and onset of late AIDS showed that children with the rs1801157 GA genotype and the R5X4 viral strain were at higher risk (OR = 8.00, 95% CI 1.23–52.25; p = 0.030) than children with the rs1801157 GG genotype and an R5 virus (Table 4). Of note, children with R5X4 variants and the rs1801157 GA genotype had a ten-fold higher risk of developing late AIDS (OR = 10.00, 95% CI 1.30–78.00; p = 0.028) than children with the rs1801157 GG genotype and a R5X4 virus, thus supporting the premise of a primarily protective role of the rs1801157 GG genotype against viruses using CXCR4 coreceptor.

**Table 4:** rs1801157 genotype and viral coreceptor usage in the onset of late AIDS.

		AIDS/N.	OR (95% CI)	p-value
Viralstrain	rs1801157 genotype			
R5R5R5X4R5X4	GGGAGGGA	3/152/72/126/9	1.001.60 (0.20–12.69)0.80 (0.11–5.77)8.00 (1.23–52.25)	0.6570.8250.030

## Discussion

Overall, these results demonstrated that rs1801157 polymorphism did not influence the susceptibility to perinatal HIV-1 infection, but strongly influenced in a time-dependent manner disease progression; children with the rs1801157 GA genotype had a 6–10 fold increased risk of developing late AIDS than children with the rs1801157 GG genotype.

To date, studies on the association between the rs1801157 genotype and HIV-1 susceptibility and disease progression have generated inconclusive results. These studies may have numerous confounding variables, such as differences in the characteristics of the study populations, the duration of follow-up, the definition of the study end-point, and the influence of antiretroviral therapy. Moreover, variation in coreceptor usage of infecting viruses and their evolution during the course of HIV-1 infection may contribute to generating different results. Our study, aimed at investigating the role of rs1801157 polymorphism in the natural history of perinatal HIV-1 infection and disease progression, was conducted in a white population with no exposure to antiretroviral prophylaxis or therapeutic interventions. Furthermore, in a subset of the studied population viral coreceptor usage was defined.

In our study, the rs1801157 genotypes were similarly distributed among infants born HIV-1-infected and HIV-1-uninfected. In agreement with our results, a recent meta-analysis reported no significant association between the rs1801157 genotype and HIV-1 susceptibility in any genetic model [47]. Notably, most of the children in developed countries acquired infection around the time of delivery [37], [48] and the transmitted viruses were predominantly of the R5 phenotype [49], [50]. In agreement with this previous finding, 94% of studied children had an R5 virus at birth. Although a selection of transmitted variants cannot be excluded [51], [52], this predominance of R5 isolates may explain the lack of a role of the rs1801157 SNP in perinatal HIV-1 infection.

*SDF1* plays a strong role in disease progression. Notably, this role is time-dependent and selectively involves the onset of late AIDS. Conversely to adults, a fraction of perinatally HIV-1-infected children develop early AIDS within the first 24 months of age [53], [54]. In this study, as well as in previous studies [5], [17], [55], most early AIDS were associated with an R5 virus. Polymorphisms in the *CCR5* promoter gene, such as the P1/P1 haplotype, may influence CCR5 expression and thus disease progression linked to R5 viruses [16]. This association may explain why genetic variants of the *CCR5*, but not the rs1801157 SNP, impact the risk of early AIDS.

In contrast, most of the late AIDS cases were associated with R5X4 viruses. While the role of P1/P1 was lost, the rs1801157 SNP acquired a strong influence, and its GA genotype was associated with a significantly higher risk of late AIDS than the GG genotype. It has been suggested that the rs1801157 A allele upregulates plasma chemokine levels [22], [23]; upregulation of SDF1 levels in rs1801157 AA and GA patients may prevent the emergence of more pathogenic R5X4 strains, thus contributing to the slowing of disease progression [24]. Nonetheless, other studies did not correlate the rs1801157 A allele with chemokine production [27], [56] and one study demonstrated that subjects with the rs1801157 A allele were also more likely to have detectable X4-tropic viruses [57]; the increased frequency of X4 viruses in subjects carrying the rs1801157 A allele may explain the observed adverse effect that this allele had on HIV-1 disease [28]–[30]. In our study we found no relationship between the rs1801157 genotype and emergence of CXCR4-usage viral strains. However, our study indicated that the impact of the rs1801157 SNP was linked to coreceptor usage of infecting viruses. In particular, while in children with the R5 virus, a GG or GA genotype did not influence the course of disease, in children with R5X4 viruses, those with the rs1801157 GA genotype had a 10-fold higher risk for developing late AIDS than those with the rs1801157 GG genotype.

These observations could have important implications for the clinical treatment of HIV-1-infected children. The discovery of CCR5 and CXCR4 as critical coreceptors for HIV-1 entry [1], [2], [4], coupled with the observation that patients heterozygous for *CCR5*Δ32 have a delayed disease progression [58], indicated that pharmacological blockade of the gp120-CCR5 interaction could be an effective strategy for inhibiting HIV-1 infection. Currently, entry inhibitors hold considerable potential for the treatment of HIV-1 infection, particularly in patients harbouring viruses resistant to traditional ART regimens with reverse transcriptase and protease inhibitors. Maraviroc, the first CCR5 inhibitor [59], is active against R5 viral strains [60]. Viruses using CXCR4 have become the dominant circulating strains in patients harboring R5X4 viruses prior to therapy [61]. The emergence of X4 strains as a result of CCR5 antagonist therapy, and the consequences on disease progression will need to be closely monitored. Data now available suggested that CCR5 antagonists might be more effective if utilized to treat patients at earlier stages of disease [62]-[65]. With this perspective, our study could be important in targeting therapy with CCR5 blockers in particular patients. Since our results suggest that the rs1801157 GA genotype is associated with a higher risk for developing AIDS, and that this effect is linked to the presence of viral strains using CXCR4, these patients may not be ideal candidates for a CCR5 inhibitor-based therapeutic regimens.
